# Exploring the role of immune cell and inflammatory cytokines in the development of rosacea

**DOI:** 10.1097/MD.0000000000046719

**Published:** 2025-12-19

**Authors:** Yang Xu, Juhua Zhao

**Affiliations:** a Department of Dermatology, Beijing Anzhen Nanchong Hospital of Capital Medical University, Nanchong Central Hospital, Nanchong, Sichuan Province, People’s Republic of China.

**Keywords:** immune cells, inflammatory cytokines, Mendelian randomization, rosacea

## Abstract

Immune cells and inflammatory cytokines collectively contribute to the pathogenesis of rosacea. However, the causality between immune cells, inflammatory cytokines and rosacea remains unclear. This study employed Mendelian randomization analysis to investigate the causality among 731 immune cells, 91 inflammatory cytokines, and rosacea. The inverse variance weighted, Mendelian randomization Egger, simple mode, weighted median, and weighted mode analyses were implemented to investigate causality. Sensitivity analyses were conducted to evaluate reliability of findings. Our research results indicated that 3 immune cells can increase risk of rosacea, 4 immune cells can decrease risk of rosacea. Among them, transitional (TR) B cell %lymphocyte (odds ratio [OR] = 1.170, 95% confidence interval [CI] = 1.001–1.368), CD25 on IgD− CD24− B cell (OR = 1.151, 95% CI = 1.013–1.307), and HLA DR on CD14− CD16+ monocyte (OR = 1.136, 95% CI = 1.007–1.281) increased risk of rosacea. Central memory CD4− CD8− T cell absolute count (OR = 0.952, 95% CI = 0.889–1.019), CCR7 on naive CD4+ T cell (OR = 0.928, 95% CI = 0.866–0.995), CD14+ CD16− monocyte absolute count (OR = 0.915, 95% CI = 0.857–0.977), and CD62L− myeloid dendritic cell (mDC; OR = 0.928, 95% CI = 0.862–0.999) decreased risk of rosacea. Meanwhile, the findings revealed that C-X-C motif chemokine 11 (CXCL11) levels (OR = 1.265, 95% CI = 0.985–1.625) and T-cell surface glycoprotein CD6 isoform levels (OR = 1.372, 95% CI = 1.069–1.761) increased risk of rosacea, while monocyte chemoattractant protein-1 levels (OR = 0.772, 95% CI = 0.621–0.960) and programmed cell death 1 ligand 1 levels (OR = 0.739, 95% CI = 0.557–0.981) decreased risk of rosacea. This study establishes a theoretical framework for comprehensively investigating the relationships between immune cells, inflammatory cytokines, and rosacea. It also offers valuable references for identifying novel therapeutic targets for rosacea.

## 1. Introduction

Rosacea is a prevalent chronic inflammatory skin disorder characterized by episodic flushing, persistent erythema, telangiectasia, papules, and pustules on the face.^[1,2]^

Rosacea can be divided into 3 types: erythematotelangiectatic rosacea, papulopustular rosacea, and phymatous rosacea.^[3,4]^ Rosacea can also adversely impact patients’ physical and mental health, potentially resulting in feelings of inferiority, depression, and anxiety.^[5,6]^ Furthermore, rosacea may be associated with various systemic diseases, including inflammatory bowel disease, rheumatoid arthritis, and coronary artery disease.^[7,8]^ The global prevalence of rosacea is approximately 5.46%.^[9]^ The pathogenesis of rosacea remains incompletely understood and is primarily associated with complex interactions among genetic factors, immune dysregulation, neurovascular dysregulation, microbial presence, and environmental influences.^[10,11]^

Immune cells play a crucial role in the pathogenesis of rosacea. In patients with rosacea, skin lesions exhibit a significant increase in the infiltration of macrophages, mast cells, regulatory T cells, and resting dendritic cells.^[12,13]^ These cells exacerbate the inflammatory response of skin lesions by releasing various inflammatory mediators, including antimicrobial peptides, vascular endothelial growth factor, interferon-γ, tumor necrosis factor, matrix metalloproteinases, and interleukin (IL)-17.^[14,15]^ Mast cells are intricately linked to neurogenic inflammation.^[16]^ Neuropeptides (NPs) activate mast cells, resulting in degranulation and the subsequent release of histamine, tryptase, and tumor necrosis factor-α. This cascade of events promotes inflammation and contributes to flushing and erythema in the skin.^[17]^

Inflammatory cytokines are also closely associated with the pathogenesis of rosacea. A clinical study showed that serum chemokine levels in patients with rosacea are significantly higher than those in healthy control groups.^[18]^ The expression of C-X-C motif chemokine ligand 8 (CXCL8) is elevated in patients with rosacea.^[19]^ A cathelicidin LL37, antimicrobial peptide induces keratinocytes to release CXCL8, a key chemokine in rosacea.^[20]^ Immune cells and inflammatory cytokines collectively contribute to the pathogenesis of rosacea. However, the causal relationship between immune cells, inflammatory cytokines and rosacea remains unclear.

Mendelian randomization (MR) is a statistical method that employs genetic variations as instrumental variables (IVs) to estimate the causal effect of an exposure on an outcome.^[21]^ MR analysis can circumvent potential confounding factors and demonstrate a significant causal association.^[22]^ This study is the first to explore the causality 731 immune cells, 91 inflammatory cytokines, and rosacea through MR analysis, providing novel directions into the immune-inflammatory mechanisms and treatment of rosacea.

## 2. Materials and methods

### 2.1. Study design

**Figure 1.**
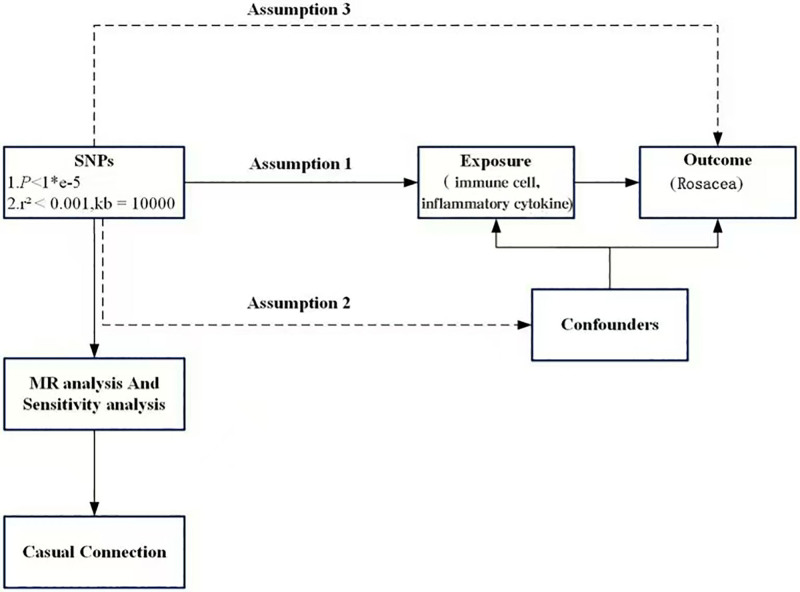
Flowchart of MR analysis. MR = Mendelian randomization.

The data were derived from the summary statistics of genome-wide association studies (GWAS). This analysis necessitated the fulfillment of 3 primary assumptions: exposure factors are strongly correlated with IVs; there are no known confounding factors associated with either exposure or outcome; and IVs only affect outcomes when they are associated with exposure factors (Fig. 1).^[23]^

### 2.2. Data sources

The data concerning immune cell phenotypes were sourced from the GWAS database (GCST0001391–GCST0002121).^[24]^ The data on 91 inflammatory cytokines can be found in a GWAS involving 14,824 subjects of European population.^[25]^ The GWAS data of rosacea were sourced from the FinnGen database, including 1195 cases and 2,11,139 controls (finn-b-L12_ROSACEA).

### 2.3. Selection of IVs

In this study, a significance threshold of *P* < 1.0 × 10^−5^ was set to screen out single-nucleotide polymorphisms (SNPs) that were associated with immune cells and inflammatory cytokines. Furthermore, the analysis of linkage disequilibrium was conducted using the following threshold (*r*² < 0.001, kb = 10,000).^[26]^ The palindromic SNPs were excluded, and the IVs was assessed using the *F*-statistic (*F* > 10) to ensure that the exposure factors were strongly related to the obtained IVs.^[27]^

### 2.4. Statistical analysis

We used the inverse variance weighted, MR-Egger, simple mode, weighted median, and weighted mode analyses for MR analysis. The inverse variance weighted method was used as a primary analytical method to evaluate the causal association. A *P*-value < .05 is considered to indicate statistically significant results. All analyses were performed using R software (version 4.4.1) and the TwoSampleMR package (version 0.6.8).

### 2.5. Sensitivity analysis

Given that a single IV can influence multiple traits simultaneously and heterogeneity may be present within the data, multiple sensitivity analyses are employed to ensure the robustness and reliability of MR results. The Cochran’s *Q* test was used to identify heterogeneity.^[28]^ MR pleiotropy residual sum and outlier (MR-PRESSO) is a statistical method for MR analysis, aiming to detect and correct the bias in the results caused by pleiotropy in IVs. The MR-Egger intercept test and the MR-PRESSO method were employed to detect the presence of horizontal pleiotropy.^[29]^ A *P*-value > .05 is considered to indicate that there is no heterogeneity in the data.

The leave-one-out method is employed to assess the stability of the finds. We investigated the impact of individual SNPs on the MR results by sequentially removing each SNP.

## 3. Results

### 3.1. Causal effect of immune cells on rosacea

**Figure 2.**
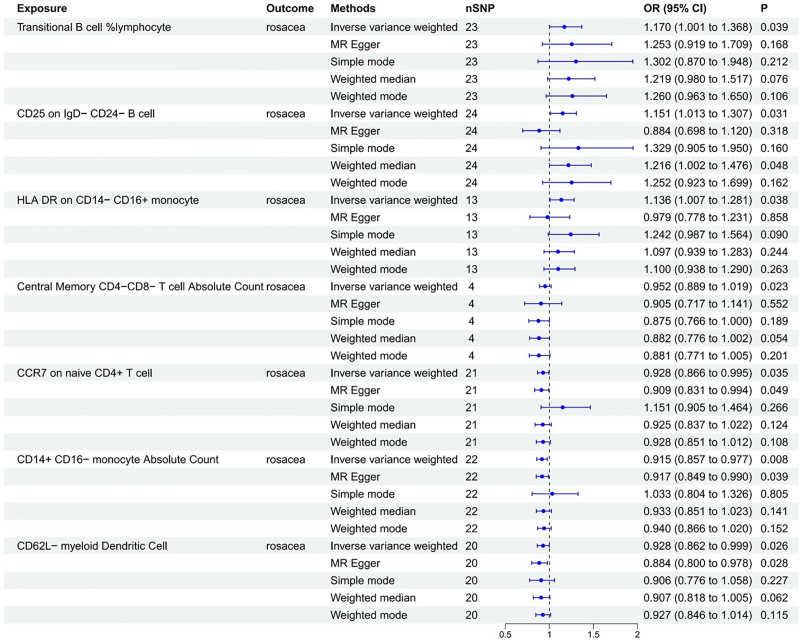
Association of immune cells with the risk of rosacea in MR analyses. CI = confidence interval, MR = Mendelian randomization, OR = odds ratio.

Our analysis identified 7 types of immune cells that are causally associated with rosacea, comprising 1 risk factor and 4 protective factors. Our research findings revealed that transitional (TR) B cell %lymphocyte (OR = 1.170, 95% CI = 1.001–1.368), CD25 on IgD− CD24− B cell (OR = 1.151, 95% CI = 1.013–1.307), and HLA DR on CD14− CD16+ monocyte (OR = 1.136, 95% CI = 1.007–1.281) show a positive correlation with the risk of rosacea, while central memory CD4− CD8− T cell absolute count (AC; OR = 0.952, 95% CI = 0.889–1.019), CCR7 on naive CD4+ T cell (OR = 0.928, 95% CI = 0.866–0.995), CD14+ CD16− monocyte AC (OR = 0.915, 95% CI = 0.857–0.977), and CD62L− myeloid dendritic cell (mDC; OR = 0.928, 95% CI = 0.862–0.999) show a negative correlation with the risk of rosacea (Fig. 2).

### 3.2. Causal effect of inflammatory cytokines on rosacea

**Figure 3.**
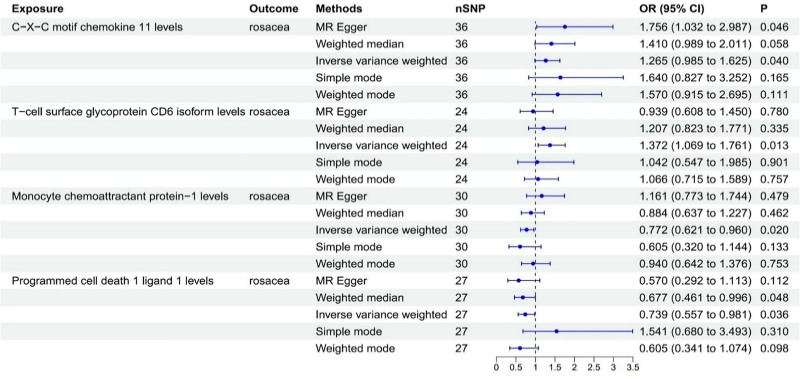
Association of inflammatory cytokines with the risk of rosacea in MR analyses. CI = confidence interval, MR = Mendelian randomization, OR = odds ratio.

Our analysis indicated that 4 inflammatory cytokines are causally associated with rosacea, comprising 2 risk factor and 2 protective factors. Our research findings revealed that CXCL11 levels (OR = 1.265, 95% CI = 0.985–1.625) and T-cell surface glycoprotein CD6 isoform levels (OR = 1.372, 95% CI = 1.069–1.761) show a positive correlation with the risk of rosacea, while monocyte chemoattractant protein-1 (MCP-1) levels (OR = 0.772, 95% CI = 0.621–0. 960) and programmed cell death 1 ligand 1 (PD-L1) levels (OR = 0.739, 95% CI = 0.557–0.981) show a negative correlation with the risk of rosacea (Fig. 3).

### 3.3. Sensitivity analysis

The MR-Egger intercept test and MR-PRESSO method indicated no significant horizontal pleiotropy (*P* > .05). The Cochran’s *Q* test results indicated the absence of heterogeneity (*P* > .05; Tables S1–S2, Supplemental Digital Content, https://links.lww.com/MD/Q981). The leave-one-out analysis revealed that no SNPs exerted a substantial influence on the causal association, thereby reinforcing the robustness of the findings. The scatter plots and funnel plots further verified the reliability of the results (Figs. S1–S6, Supplemental Digital Content, https://links.lww.com/MD/Q981).

## 4. Discussion

The results indicated significant associations between 7 types of immune cells and the pathogenesis of rosacea. TR B cell %lymphocyte, CD25 on IgD− CD24− B cell, and HLA DR on CD14− CD16+ monocyte are risk factors for the onset of rosacea, while central memory CD4− CD8− T cell AC, CCR7 on naive CD4+ T cell, CD14+ CD16− monocyte AC, and CD62L − mDC are protective factors for the onset of rosacea. Furthermore, CXCL11 levels and T-cell surface glycoprotein CD6 isoform levels are risk factors for rosacea, while MCP-1 levels and PD-L1 levels are protective factors for rosacea.

In the skin lesions of patients with rosacea, 10% to 20% of the inflammatory cell infiltrates are composed of CD20 B cells, accompanied by a small amount of plasma cell infiltration.^[30]^ Transcriptome analysis revealed that the RNA expression level of the B-cell marker CD20 was significantly elevated in patients with rosacea. Additionally, immunohistochemical analysis demonstrated a substantial increase in plasma cells in rosacea.^[12]^ CD25, a critical subunit of the IL-2 receptor, enhances signal transduction upon binding to IL-2. It is expressed on B cells, where it promotes their proliferation and differentiation, and may possess immunomodulatory functions.^[31,32]^ This study identified that the Transitional B cell % lymphocyte and CD25 on IgD− CD24− B cell are risk factors for the development of rosacea. Nonetheless, further research is required to elucidate the correlation between these specific immune cells and the pathogenesis of rosacea.

Our study revealed HLA DR on CD14− CD16+ monocyte is a risk factor for the incidence of rosacea. CD14 and CD16 are surface markers expressed on immune cells. The CD14− CD16+ phenotype predominantly identifies monocytes, which are crucial for signal recognition and transduction.^[33,34]^ A retrospective study demonstrated that the monocyte count in the blood of patients with rosacea was significantly higher than that of healthy controls.^[35]^ Another study observed an increased proportion of classical monocytes in the peripheral blood of patients with rosacea, while the proportions of intermediate and nonclassical monocytes remained unchanged. Additionally, the proportion of classical monocytes decreased following treatment.^[36]^ HLA-DR is a key member of the major histocompatibility complex class II (MHC II) molecule family, responsible for presenting exogenous antigens and initiating the adaptive immune response mediated by CD4+ T cells.^[37,38]^ We speculate that HLA-DR plays a critical role in the pathogenesis of rosacea. However, existing research on the association between HLA-DR and rosacea remains limited, and further studies are necessary to confirm this relationship.

In patients with rosacea, the expression levels of CD4+ and CD8+ T cells are elevated, along with an upregulation of polarization genes associated with Th1 and Th17 cells. Additionally, the concentrations of IFN-γ and IL-17A in rosacea lesions are significantly increased.^[12]^ IL-17 has the capacity to induce angiogenesis and enhance the expression of the antimicrobial peptide LL-37 in human epidermal keratinocytes, thereby contributing to the pathogenesis of rosacea.^[39,40]^ The study revealed that the average percentage of CD4+ CD25+ regulatory T cells in patients with rosacea was significantly higher than that observed in patients with lupus erythematosus.^[41]^ Our study revealed central memory CD4− CD8− T cell and CCR7 on naive CD4+ T cell are protective factors for rosacea. Myeloid dendritic cells are a subtype of antigen-presenting cells that present external antigens to activate immune responses.^[42,43]^ Our study found that CD62L − mDC can reduce the incidence of rosacea. Since there is currently a lack of research on CD62L − mDC in the pathogenesis of rosacea, further studies are still needed in the future to confirm the results of this research

Chemokines are closely associated with the progression of rosacea. Research has demonstrated that CXCL8, CXCL1, and CXCL2 are significantly upregulated in patients with rosacea. These chemokines facilitate angiogenesis and promote the recruitment of neutrophils and Th17 cells.^[44]^ A clinical study identified the candidate gene CXCL11 as a potential therapeutic target for rosacea using bioinformatics methods.^[45]^ This finding aligns with our research results, which indicate that CXCL11 elevates the risk of rosacea development. Our research findings indicated that MCP-1 and PD-L1 are associated with a reduced risk of developing rosacea. MCP-1 may facilitate monocyte aggregation and could play a regulatory role in the microbiota of rosacea patients.^[46]^ PD-L1 binds to PD-1 receptors on the surface of T cells, inhibiting their activation and proliferation and thus downregulating the immune response.^[47]^ However, there remains a lack of studies investigating the association between MCP-1, PD-L1, and rosacea to validate the findings of this study.

Compared to previous studies, our research utilizes large-scale GWAS data and employs MR analysis to minimize potential confounding factors, thereby providing a more reliable causal relationship. However, this article still has a few drawbacks. First, the GWAS data are originated solely from European populations, the generalizability of the conclusion of this study to patients of other ethnicities is limited. Second, the current data do not permit stratified analyses of various rosacea subtypes. The association between various forms of rosacea and the infiltration of immune cells and inflammatory mediators requires further validation. In addition, there may be potential interactions among different immune cell that could influence the research outcomes. Therefore, it is essential to conduct a comprehensive analysis of the mechanisms of action of various immune cells in rosacea.

## 5. Conclusion

In conclusion, this study reveals the casual roles of immune cells and inflammatory cytokines in the development of rosacea based on large-scale GWAS data. This study establishes a theoretical framework for comprehensively investigating the relationships between immune cells, inflammatory cytokines, and rosacea. It also offers valuable references for identifying novel therapeutic targets for rosacea.

## Acknowledgments

The authors thank the genome-wide association study (GWAS) for sharing public data.

## Author contributions

**Conceptualization:** Yang Xu.

**Formal analysis:** Yang Xu.

**Supervision:** Juhua Zhao.

**Writing – original draft:** Yang Xu.

**Writing – review & editing:** Juhua Zhao.

## Supplementary Material


