# Supplementary material for: Exploring the role of immune cell and inflammatory cytokines in the development of rosacea
Source: Medicine (Baltimore). 2025 Dec 19;104(51):e46719. doi: 10.1097/MD.0000000000046719 (PMC12727314; doi:10.1097/MD.0000000000046719)
Supplement: Supplementary file 1 [file medi-104-e46719-s001.pdf]

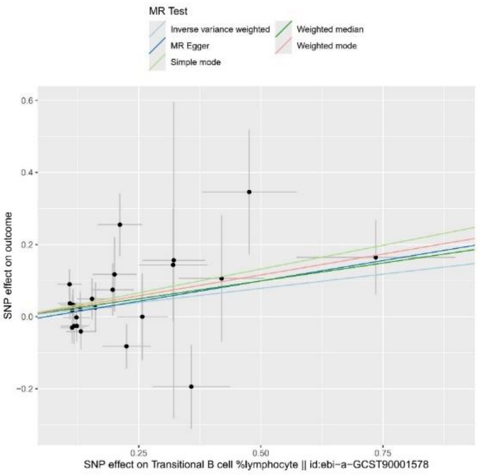
MR Test
Inverse variance weighted
MR Egger
Simple mode
Weighted median
Weighted mode
SNP effect on outcome
SNP effect on Transitional B cell %lymphocyte || id:ebi-a-GCST90001578

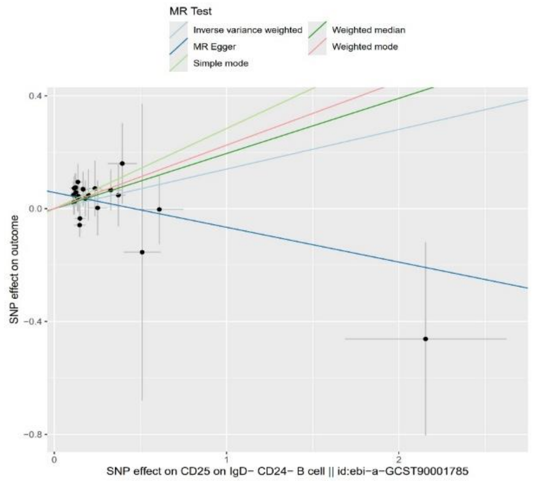
MR Test
Inverse variance weighted
MR Egger
Simple mode
Weighted median
Weighted mode
SNP effect on outcome
SNP effect on CD25 on IgD- CD24- B cell || id:ebi-a-GCST90001785

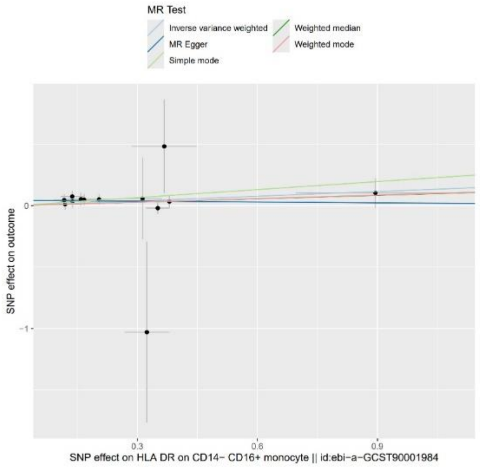
MR Test
Inverse variance weighted
MR Egger
Simple mode
Weighted median
Weighted mode
SNP effect on outcome
SNP effect on HLA DR on CD14- CD16+ monocyte || id:ebi-a-GCST90001984

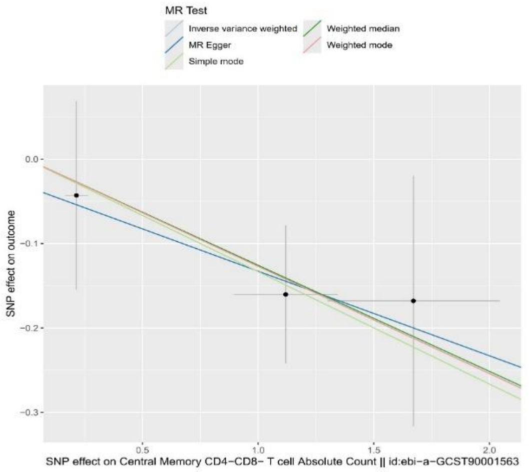
MR Test
Inverse variance weighted
MR Egger
Simple mode
Weighted median
Weighted mode
SNP effect on outcome
SNP effect on Central Memory CD4-CD8- T cell Absolute Count || id:ebi-a-GCST90001563

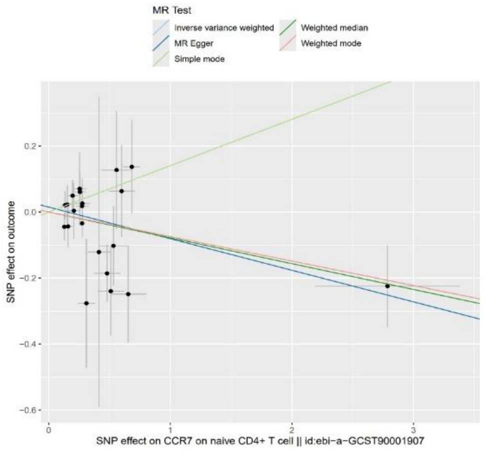
MR Test
Inverse variance weighted
MR Egger
Simple mode
Weighted median
Weighted mode
SNP effect on outcome
SNP effect on CCR7 on naive CD4+ T cell || id:ebi-a-GCST90001907

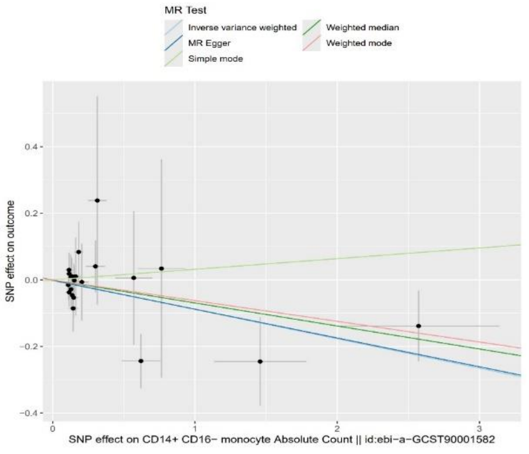
MR Test
Inverse variance weighted
MR Egger
Simple mode
Weighted median
Weighted mode
SNP effect on outcome
SNP effect on CD14+ CD16- monocyte Absolute Count || id:ebi-a-GCST90001582

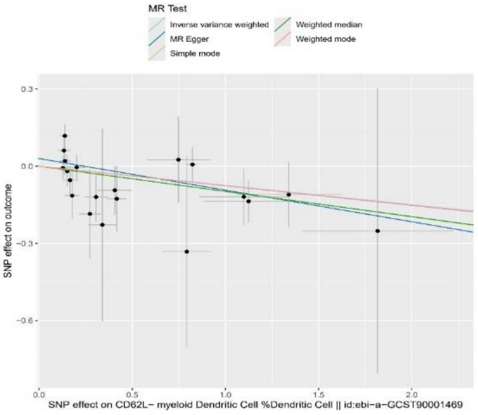


**Figure S1.** Scatter plots of the causal effect of immune cell on rosacea.

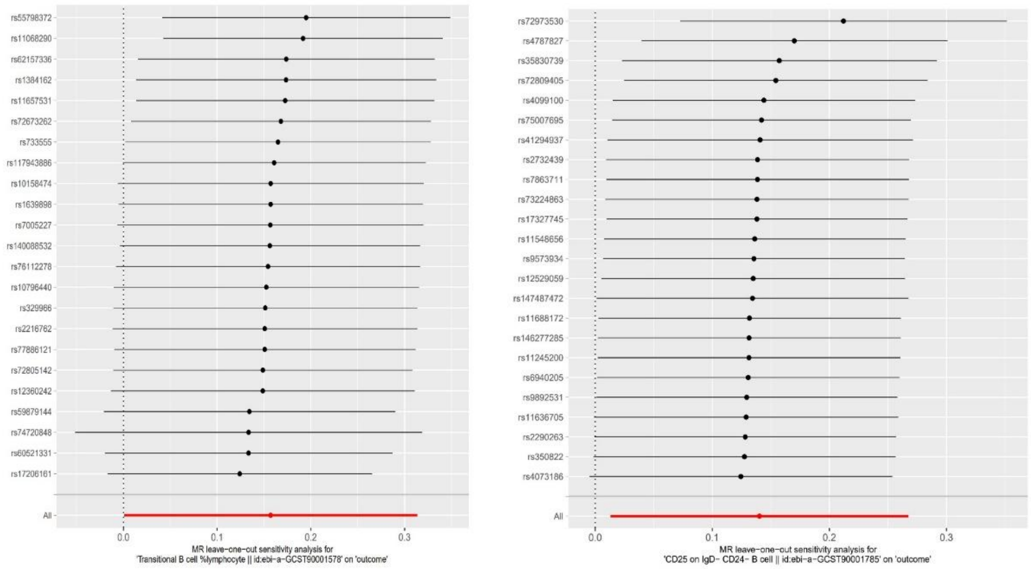

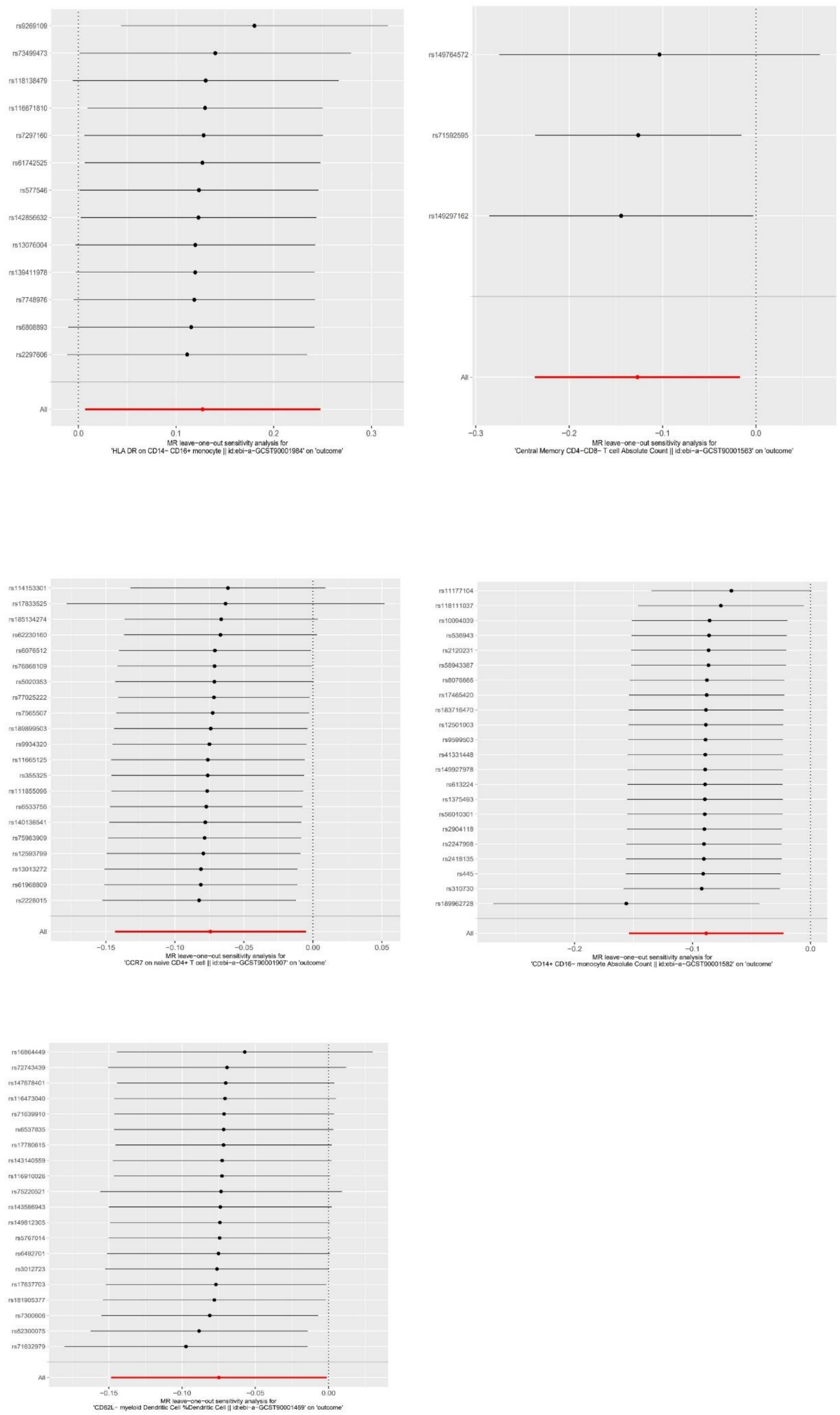


**Figure S2.** Leave-one-out analysis of the causal effect of immune cell on rosacea.

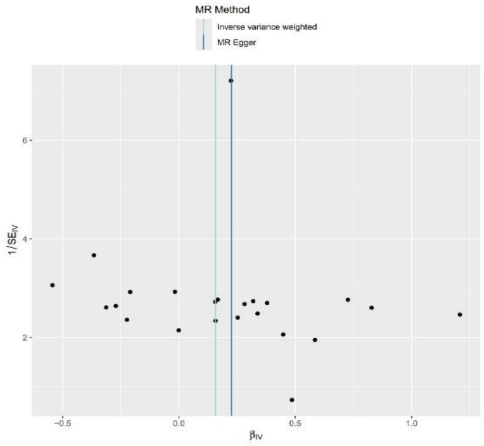
MR Method
Inverse variance weighted
MR Egger
1/SE_IV
β_IV

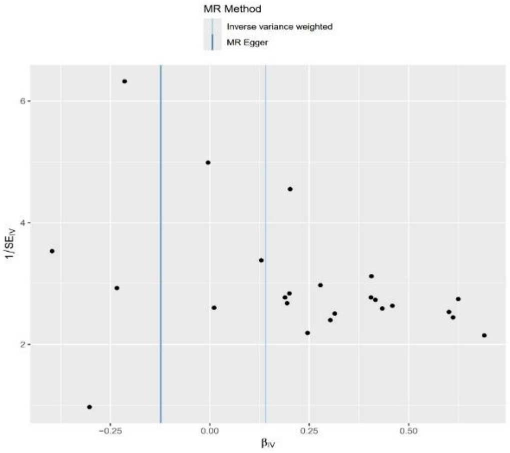
MR Method
Inverse variance weighted
MR Egger
1/SE_IV
β_IV

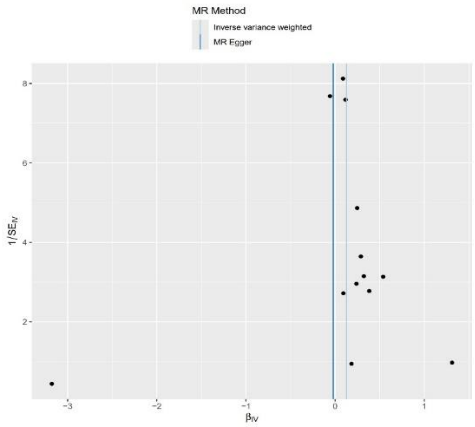
MR Method
Inverse variance weighted
MR Egger
1/SE_IV
β_IV

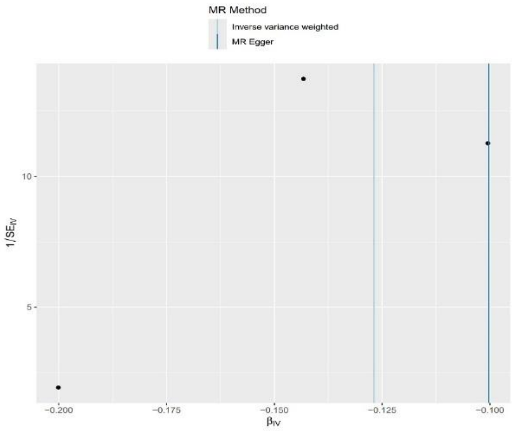
MR Method
Inverse variance weighted
MR Egger
1/SE_IV
β_IV

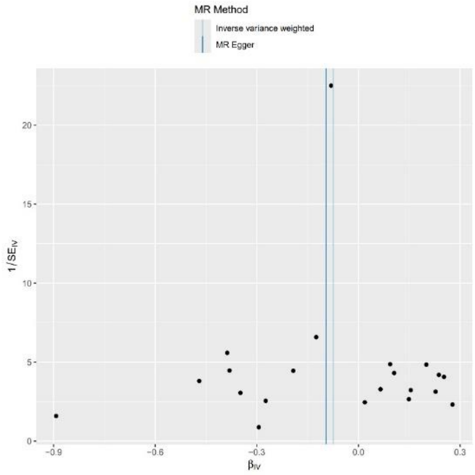
MR Method
Inverse variance weighted
MR Egger
1/SE_IV
β_IV

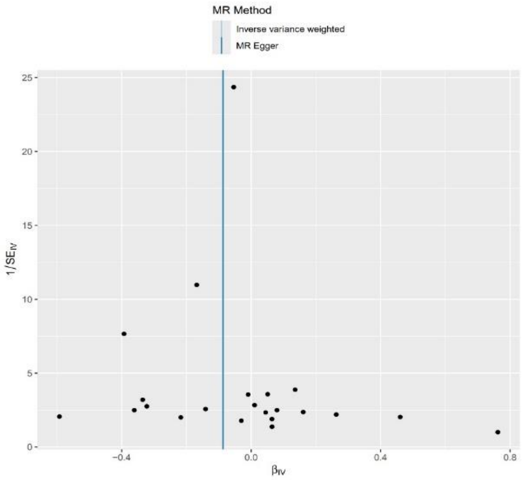
MR Method
Inverse variance weighted
MR Egger
1/SE_IV
β_IV

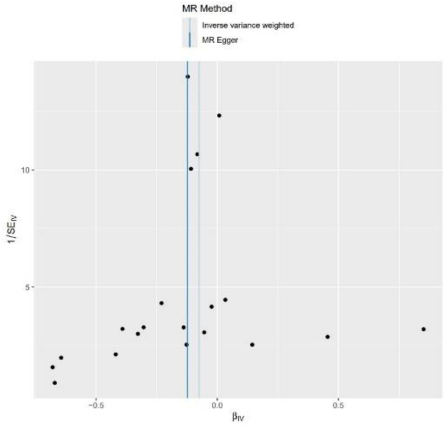


**Figure S3.** Funnel plots of the causal effect of immune cell on rosacea.

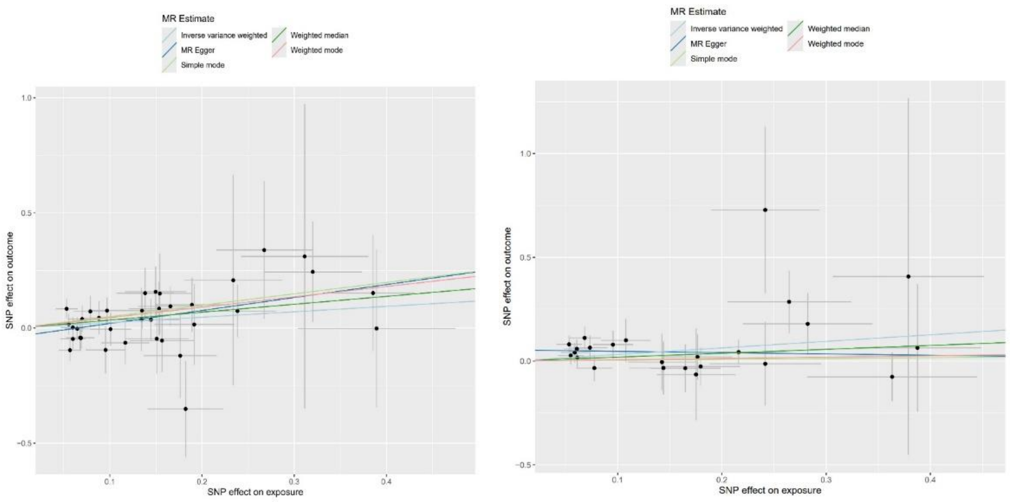

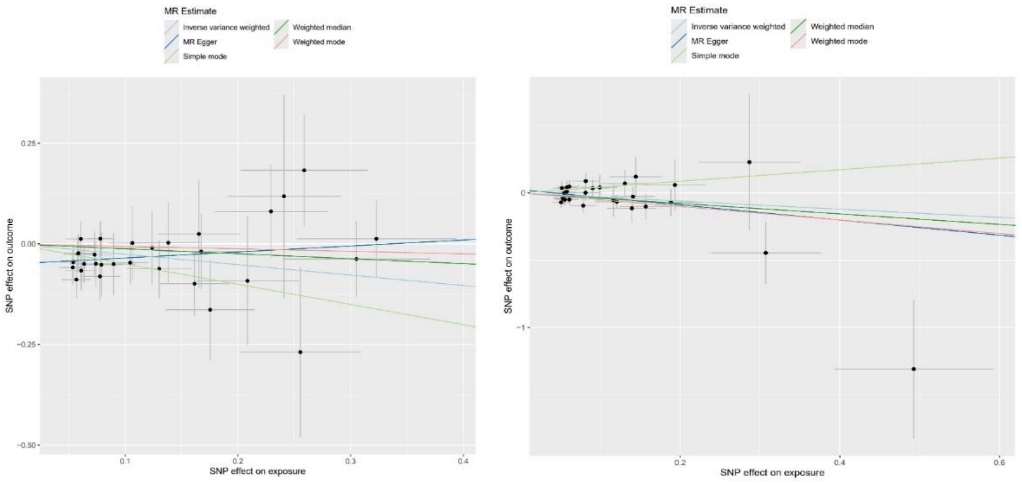


**Figure S4.** Scatter plots of the causal effect of inflammatory cytokines on rosacea.

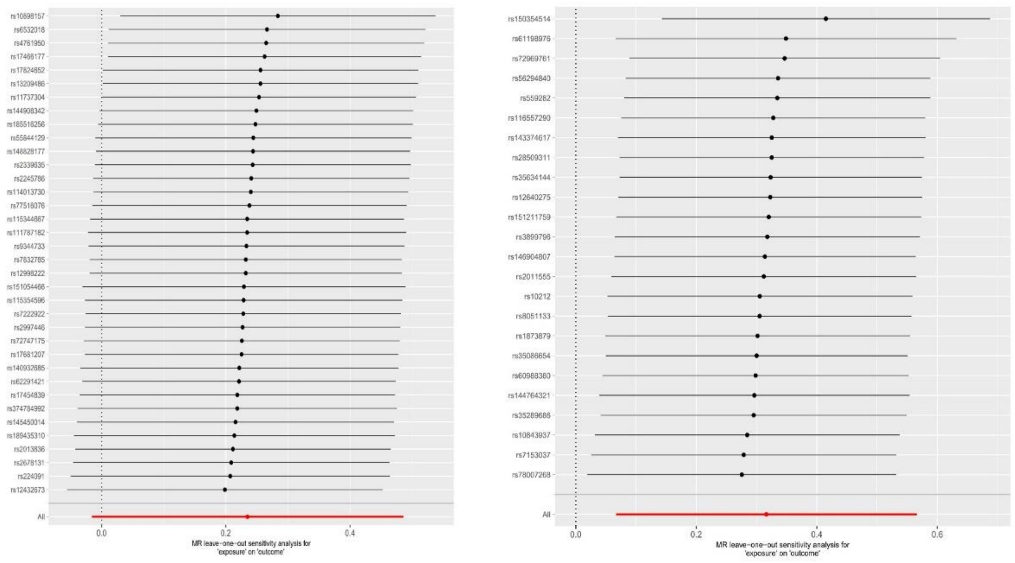

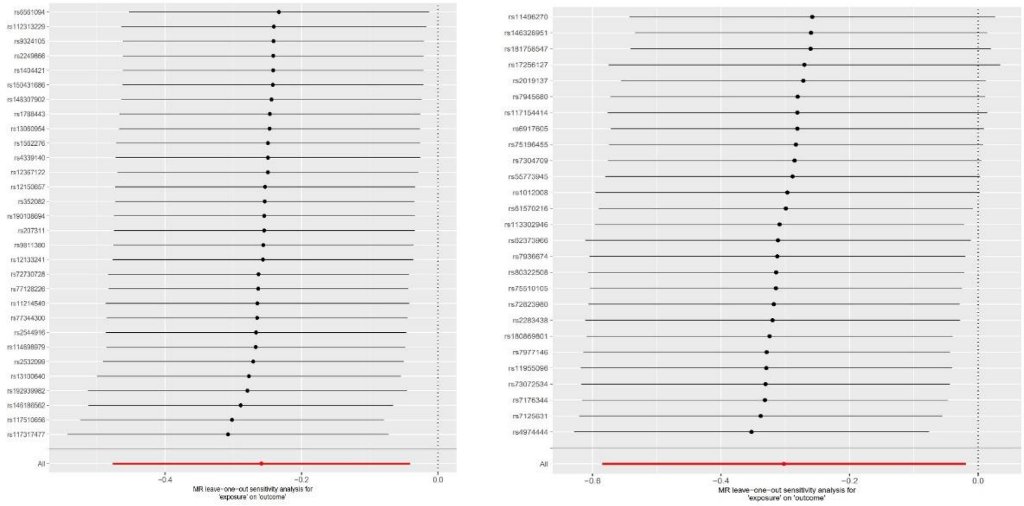


**Figure S5.** Leave-one-out analysis of the causal effect of inflammatory cytokines on rosacea.

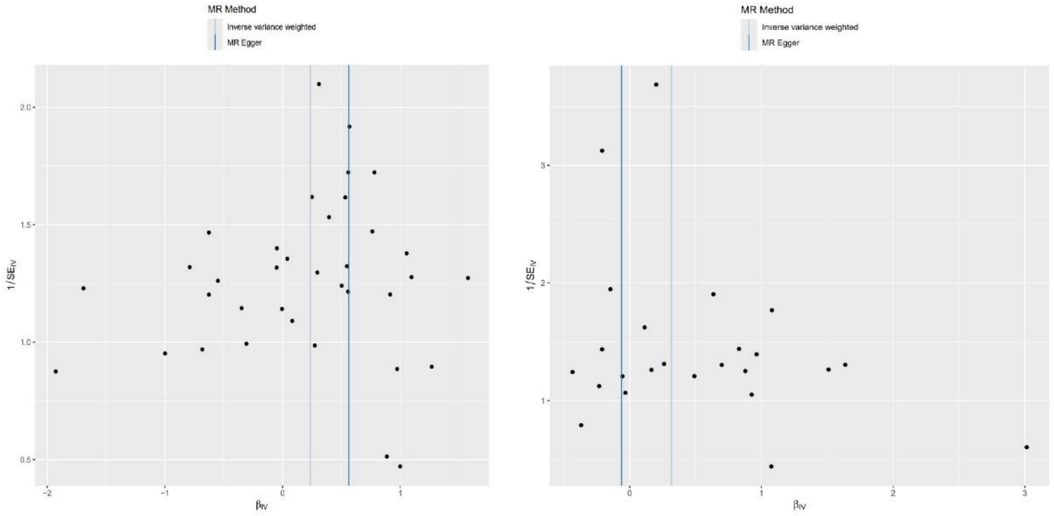

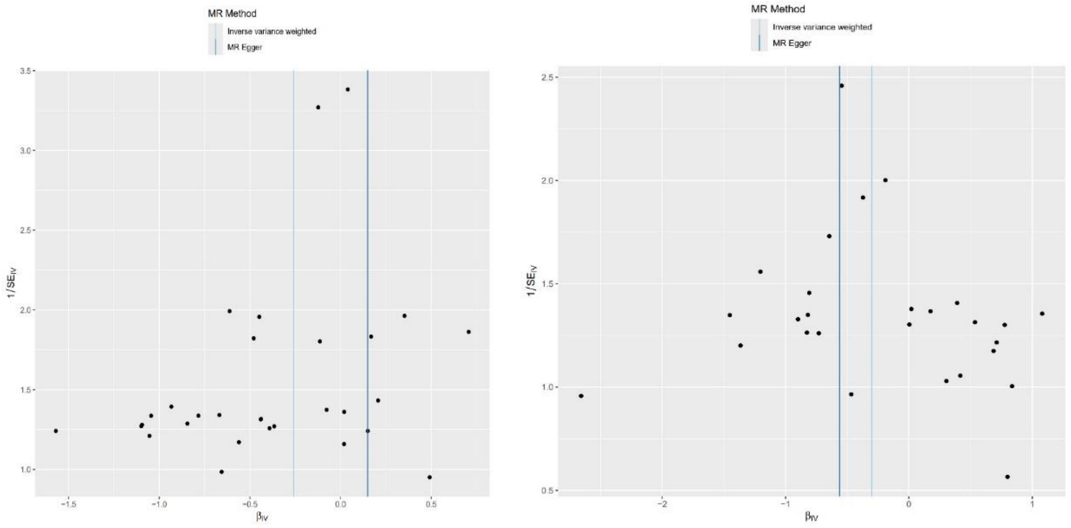


**Figure S6.** Funnel plots of the causal effect of inflammatory cytokines on rosacea.

Table S1 Sensitivity analysis immune cells on rosacea

| exposure | outcome | method | Q | Q_pval | intercept | pval | MR-PRESSO |
|---|---|---|---|---|---|---|---|
| Transitional B cell %lymphocyte | rosacea | MR Egger | 27.564 | 0.152 | -0.013 | 0.618 | 0.235 |
| | | IVW | 27.901 | 0.179 | | | |
| CD25 on IgD- CD24- B cell | rosacea | MR Egger | 13.568 | 0.916 | 0.057 | 0.056 | 0.138 |
| | | IVW | 20.289 | 0.624 | | | |
| HLA DR on CD14- CD16+ monocyte | rosacea | MR Egger | 6.722 | 0.821 | 0.042 | 0.164 | 0.152 |
| | | IVW | 8.942 | 0.707 | | | |
| Central Memory CD4-CD8- T cell Absolute Count | rosacea | MR Egger | 0.093 | 0.759 | -0.032 | 0.841 | 0.458 |
| | | IVW | 0.158 | 0.923 | | | |
| CCR7 on naive CD4+ T cell | rosacea | MR Egger | 18.948 | 0.460 | 0.014 | 0.464 | 0.527 |
| | | IVW | 19.506 | 0.489 | | | |
| CD14+ CD16- monocyte Absolute Count | rosacea | MR Egger | 14.052 | 0.827 | -0.001 | 0.928 | 0.259 |
| | | IVW | 14.060 | 0.866 | | | |
| CD62L- myeloid Dendritic Cell | rosacea | MR Egger | 17.141 | 0.513 | 0.029 | 0.191 | 0.082 |
| | | IVW | 18.986 | 0.457 | | | |

Table S2 Sensitivity analysis inflammatory cytokines on rosacea

| exposure | outcome | method | Q | Q_pval | intercept | pval | MR-PRESSO |
|---|---|---|---|---|---|---|---|
| C-X-C motif chemokine 11 | rosacea | MR Egger | 26.708 | 0.809 | -0.036 | 0.179 | 0.814 |
| | | IVW | 28.586 | 0.769 | | | |
| T-cell surface glycoprotein CD6 isoform | rosacea | MR Egger | 14.718 | 0.874 | 0.053 | 0.058 | 0.692 |
| | | IVW | 19.081 | 0.696 | | | |
| Monocyte chemoattractant protein-1 | rosacea | MR Egger | 13.120 | 0.992 | -0.049 | 0.087 | 0.930 |
| | | IVW | 18.532 | 0.932 | | | |
| Programmed cell death 1 ligand 1 | rosacea | MR Egger | 27.468 | 0.332 | 0.023 | 0.408 | 0.355 |
| | | IVW | 28.244 | 0.346 | | | |
